# In Vivo Administration of Phosphatidic Acid, a Direct Alcohol Target Rescues Fetal Growth Restriction and Maternal Uterine Artery Dysfunction in Rat FASD Model

**DOI:** 10.3390/nu16101409

**Published:** 2024-05-08

**Authors:** Joseph D. Janeski, Vishal D. Naik, Alexander L. Carabulea, Hong Jiang, Jayanth Ramadoss

**Affiliations:** 1 Department of Obstetrics and Gynecology, C.S. Mott Center for Human Growth and Development, School of Medicine, Wayne State University, Detroit, MI 48201, USA; 2 Department of Physiology, School of Medicine, Wayne State University, Detroit, MI 48201, USA

**Keywords:** alcohol, pregnancy, phosphatidic acid, FASD

## Abstract

Fetal growth restriction is a hallmark of Fetal Alcohol Syndrome (FAS) and is accompanied by maternal uterine circulatory maladaptation. FAS is the most severe form of Fetal Alcohol Spectrum Disorder (FASD), a term for the range of conditions that can develop in a fetus when their pregnant mother consumes alcohol. Alcohol exerts specific direct effects on lipids that control fundamental developmental processes. We previously demonstrated that direct in vitro application of phosphatidic acid (PA, the simplest phospholipid and a direct target of alcohol exposure) to excised uterine arteries from alcohol-exposed rats improved vascular function, but it is unknown if PA can rescue end organ phenotypes in our FASD animal model. Pregnant Sprague-Dawley rats (*n* = 40 total dams) were gavaged daily from gestational day (GD) 5 to GD 19 with alcohol or maltose dextrin, with and without PA supplementation, for a total of four unique groups. To translate and assess the beneficial effects of PA, we hypothesized that in vivo administration of PA concomitant with chronic binge alcohol would reverse uterine artery dysfunction and fetal growth deficits in our FASD model. Mean fetal weights and placental efficiency were significantly lower in the binge alcohol group compared with those in the control (*p* < 0.05). However, these differences between the alcohol and the control groups were completely abolished by auxiliary in vivo PA administration with alcohol, indicating a reversal of the classic FAS growth restriction phenotype. Acetylcholine (ACh)-induced uterine artery relaxation was significantly impaired in the uterine arteries of chronic in vivo binge alcohol-administered rats compared to the controls (*p* < 0.05). Supplementation of PA in vivo throughout pregnancy reversed the alcohol-induced vasodilatory deficit; no differences were detected following in vivo PA administration between the pair-fed control and PA alcohol groups. Maximal ACh-induced vasodilation was significantly lower in the alcohol group compared to all the other treatments, including control, control PA, and alcohol PA groups (*p* < 0.05). When analyzing excitatory vasodilatory p1177-eNOS, alcohol-induced downregulation of p1177-eNOS was completely reversed following in vivo PA supplementation. In summary, these novel data utilize a specific alcohol target pathway (PA) to demonstrate a lipid-based preventive strategy and provide critical insights important for the development of translatable interventions.

## 1. Introduction

Consuming alcohol during gestation can significantly impact fetal development, leading to Fetal Alcohol Spectrum Disorder (FASD) [1,2]. FASD comprises of a spectrum of physical and neurobehavioral effects, which include lower birth weights, shorter-than-average heights, and other developmental impairments [3,4]. The most severe of the disorders on this spectrum is Fetal Alcohol Syndrome (FAS), a term that describes specific clinically-diagnosable morphological and functional manifestations, including intrauterine growth restriction (IUGR) [3,5]. Gestational use of alcohol is estimated to be ~9.8% globally [6,7], with use during early pregnancy recently estimated to be 49.7% in eight metropolitan areas in the south/south-east regions of the United States [8] and binge alcohol use during pregnancy estimated at 3.1% [9]. Recent estimates range from 3.1 to 9.9% of school-aged children across the range of FASD [2,10]. There is no current cure for FASD, however, it has been shown that early intervention and continuing support can help manage symptoms and difficulties [3].

IUGR is directly related to compromised uterine blood flow, increased uterine artery resistance, and arterial remodeling [11,12]. Total volumetric blood flow through the uterine artery is significantly lower in cases of IUGR, and the primary uterine artery along with its proximal segments exhibit less dilation than in normal pregnancies [13]. The uterine artery is a unique vasculature that undergoes major remodeling during gestation to deliver oxygen and nutrients to the developing fetus [12,14,15,16,17,18]. During a normal pregnancy, a significant pathway to induce uterine artery vasorelaxation at least in the primary uterine artery is the endothelial nitric oxide system [17,19]. We have previously demonstrated that binge alcohol exposure during pregnancy hinders normal gestational adaptations of the maternal uterine artery, specifically impeding endothelial nitric oxide synthase (eNOS)-induced vasodilation [20,21,22,23,24,25]. This insufficient vasodilation naturally leads to decreased delivery of gases as well as nutrients to both the fetal and the placental units [26,27]. The exposure protocol employed in this study mirrors reported binge alcohol consumption habits observed in pregnant people, and those admitted to emergency wards, as well as binge exposure patterns utilized in animal models of Fetal Alcohol Spectrum Disorder (FASD) [28,29,30,31].

We therefore conjectured that manipulating the vasodilatory eNOS pathway via a novel nutrient system can be an effective method of countering alcohol’s effects on fetal growth. As Phosphatidic Acid (PA) formation is directly inhibited by alcohol [32], we chose to specifically target this endogenous lipid-based nutrient. PA is involved in membrane phospholipid biosynthesis and is commonly used as an indicator of lipid sufficiency [33,34]. However, in the presence of alcohol (ethanol), a transphosphatidylation reaction is promoted, which then results in the production of Phosphatidylethanol (PEth) [35], an abnormal class of phospholipids that accumulates in cell membranes [36] which leads to a decrease in the bioavailability of PA. To confirm, we reported an increase in specific PEth isoforms in our rat FASD model [37] and a decrease in PA isoforms in the maternal serum [38]. We further directly added PA to excised uterine artery in vitro and demonstrated that the direct addition of PA ameliorates uterine artery (UA) dysfunction and reverses alcohol-induced decreased excitatory phosphorylation of vasodilatory eNOS [39], but it is unknown if in vivo PA can rescue end organ phenotypes in our FASD animal model. To translate and assess the beneficial effects of PA, we hypothesized that in vivo administration of PA concomitant with chronic binge alcohol would reverse uterine artery dysfunction and fetal growth deficits in our FASD model.

## 2. Materials and Methods

### 2.1. Treatment Groups and Alcohol/PA In Vivo Dosing Paradigm

All experimental procedures were performed as per the National Institutes of Health guidelines (Revised NIH Publication No. 85–23, 1996; US Department of Health, Education and Welfare, Bethesda, MD, USA), with approval by the Animal Care and Use Committee at Wayne State University. Timed pregnant Sprague–Dawley rats (8–12 weeks old), were purchased from Charles River (Wilmington, MA, USA) and arrived on GD 4, where they were housed in a temperature-controlled room (23 °C) with a 12:12 h light-dark cycle. The dams were acclimatized for a day before weighing and handling. The dams were then assigned into experimental groups. Four in vivo treatment groups were utilized: (1) a nutritional pair-fed control group (control), that served as a control for nutrition and for the gavage procedure. To control for the calories derived from alcohol, these pair-fed control rats were administered isocaloric maltose-dextrin (once-daily) via orogastric gavage. (2) A binge alcohol group (alcohol), where dams were acclimatized with a once-daily gavage (orogastric) of 4.5 g/kg ethanol (22.5% weight/volume; peak blood alcohol concentration (BAC), 216 mg/dL) from gestational days (GD) 5–10, and progressed to a 6 g/kg alcohol from GD 11 to 19 (28.5% weight/volume; peak BAC, 289 mg/dL) [22,40]. The exposure paradigm utilized in this study was carefully modeled after published gestational alcohol consumption patterns in humans as well as several FAS animal models [29,41,42]. (3) An in vivo phosphatidic acid (PA) control group (control PA), to control for the in vivo PA supplement. These dams were similar to the nutritional pair-fed control group except that they were administered PA via an intragastric gavage along with the maltose-dextrin. (4) A binge alcohol in vivo PA group (alcohol PA), that received alcohol via intragastric gavage similar to those in the binge alcohol group along with the PA supplement. Daily PA doses were calculated based on the dam’s weight at the time of administration (PA (µL) = 0.2 × dam weight (g) GD 5–10; PA (µL) = 0.2105 × weight (g) GD 11–19). Intake of food in the alcohol treatment group was measured, and an equivalent amount of food was given to the pair-fed control dams to account for additional nutritional factors. Rats were euthanized by decapitation while under isoflurane anesthesia.

### 2.2. Maternal and Fetal Weight Measurements

Maternal weights and fetal weights (pair-fed control, *n* = 5; alcohol, *n* = 5; control PA, *n* = 5; alcohol PA, *n* = 5) were measured on GD 20, one day after the last treatment on GD 19, following euthanasia.

### 2.3. Reagent Preparation

HEPES-Bicarbonate Solution (HBS) (NaCl 130 mM; MgSO_4.7_H_2_O 2.5 mM; KCl 4 mM; CaCl_2_ 2.4 mM; NaHCO_3_ 4.05 mM; KH_2_PO_4_ 1.18 mM; HEPES 10 mM; EDTA 0.024 mM; Glucose 6 mM; pH 7.4) was prepared fresh on the day of experiment. PA (#840857C, Avanti Polar Lipids) was suspended in 1% Bovine Serum Albumin (BSA) to make a 10^−2^ M stock solution, then prepared in two separate 2.25% and 2.85% solutions, aliquoted for dosage. Acetylcholine (ACh) and Thromboxane (Tbx) stocks were prepared using standard procedures in HBS.

### 2.4. Arteriography

Following euthanasia, the entirety of the uterus was excised and immediately placed in ice-cold HBS for pressure arteriography experiments. Uterine artery functional assessments were performed following uterine artery isolation as described previously [20,23,43,44]. Briefly, the uterine horn was transferred to a 200 mm petri dish with solidified Sylgard to facilitate tissue isolation and cleaning in ice-cold HBS. A 3–5 mm segment of the primary uterine artery was dissected between arterial bifurcations from the approximate center of the uterine horn. Surrounding adipose and connective tissues were carefully removed from the uterine artery segment. Through the dual-chamber setup, arterial segments were mounted simultaneously from a treatment (alcohol or alcohol PA) and the respective control (control or control PA) groups, ensuring identical treatment per experiment. The dual-chamber, with mounted vessels, was put in a closed enclosure with 37 °C ambient temperature, and the cannulation setup was completed to allow continuous circulation of a pre-warmed HBS bath. Intramural pressure was increased to 60 mm Hg until the vessels exhibited a myogenic tone (~15–20 min). Following equilibration, the circulation buffer was changed to fresh HBS warmed to 37 °C. Intramural pressure was then increased to mimic in vivo pressures at 90 mm Hg, at which pressure the ACh concentration responses were measured. Vessels were pre-constricted with 10^−6^ M Tbx as determined previously [20]. Vessels that failed to demonstrate myogenic tone or did not respond to Tbx were discarded. The above treatment was followed by the administration of three-fold increasing concentrations of ACh from 10^−10^ M up to 10^−5^ M. Vascular response was recorded as previously described using Ionwizard software version 6.6 (Ionoptix LLC, Westwood, MA, USA) for at least 5 min, or until arterial diameter stabilized.

### 2.5. Immunoblotting

Immunoblotting was performed using standard laboratory procedures as previously described [24,45]. In a separate cohort of dams (pair-fed control, *n* = 5; alcohol, *n* = 5; control PA, *n* = 5; alcohol PA, *n* = 5), following euthanasia, the uterine arteries were isolated by separating the vein, and cleaning the adipose tissues in HBS, before flash freezing for immunoblot analysis. Tissues were first homogenized using a 4 °C cooled bead homogenizer (Benchmark Scientific, Sayreville, NJ, USA) and then quantified using BCA protein quantification assay. Next, 20 µg of the uterine artery sample protein was then loaded on to 4–20% mini-protean TGX gels (Bio-rad, Hercules, CA, USA). Following transfer to a PVDF membrane, P-Ser1177 eNOS (Novus Biologicals, Centennial, CO, USA), total eNOS (BD Biosciences, Franklin Lakes, NJ, USA), and β-Actin (Sigma Aldrich, St. Louis, MI, USA) were probed. Densitometry analysis was performed using AzureSpot (Azure Biosystems, Dublin, CA, USA).

### 2.6. Immunofluorescence

Immunofluorescence assessments were performed using previously published protocols [46,47]. In brief, maternal uterine arteries were sectioned at 8 μm with a Leica cryostat (CM1860, Leica Biosystems, Buffalo Grove, IL, USA). Sections were subsequently fixed with ice-cold methanol (30 min, −20 °C), and then rinsed in PBS, and incubated in 10% normal serum (60 min). This was followed by incubation with (1:100; p1177-eNOS; Cell Signaling and 1:250; total eNOS, BD Biosciences) primary antibody overnight at 4 °C in a humidified chamber. The sections were then incubated with goat anti-rabbit IgG secondary antibody (Alexa Fluor 488, Invitrogen, Carlsbad, CA, USA), for 1 h at room temperature. Digital images were captured with an Olympus BX63 stereomicroscope that was equipped with U-HGLGPS fluorescent light source, ORCA-Flash 4.0 LT camera, Hamamatsu Photonics (Hamamatsu, Japan), and Olympus cellSens Dimension software Version 3.2 (Olympus, Tokyo, Japan).

### 2.7. Statistics

Maternal weight and fetal weight (unit of analysis is a dam or litter) were analyzed using two-way mixed ANOVA with alcohol as the between factor and PA as the within factor. Normality was tested using the Shapiro-Wilk normality test where appropriate. Uterine vascular response to ACh was analyzed using two-way ANOVA, followed by multiple comparisons using Fisher’s LSD. Data for the ACh concentration response following ANOVA were reported as mean ± SEM. Non-linear regression curve fit was performed using a three-parameter equation, Y = Baseline + (Max Response-Baseline)/1 + 10 ^(LogEC50-X)^ to obtain the effective concentration (EC50). The data were considered significant if the *p* value was <0.05.

## 3. Results

### 3.1. Growth Assessment

**Figure 1:**
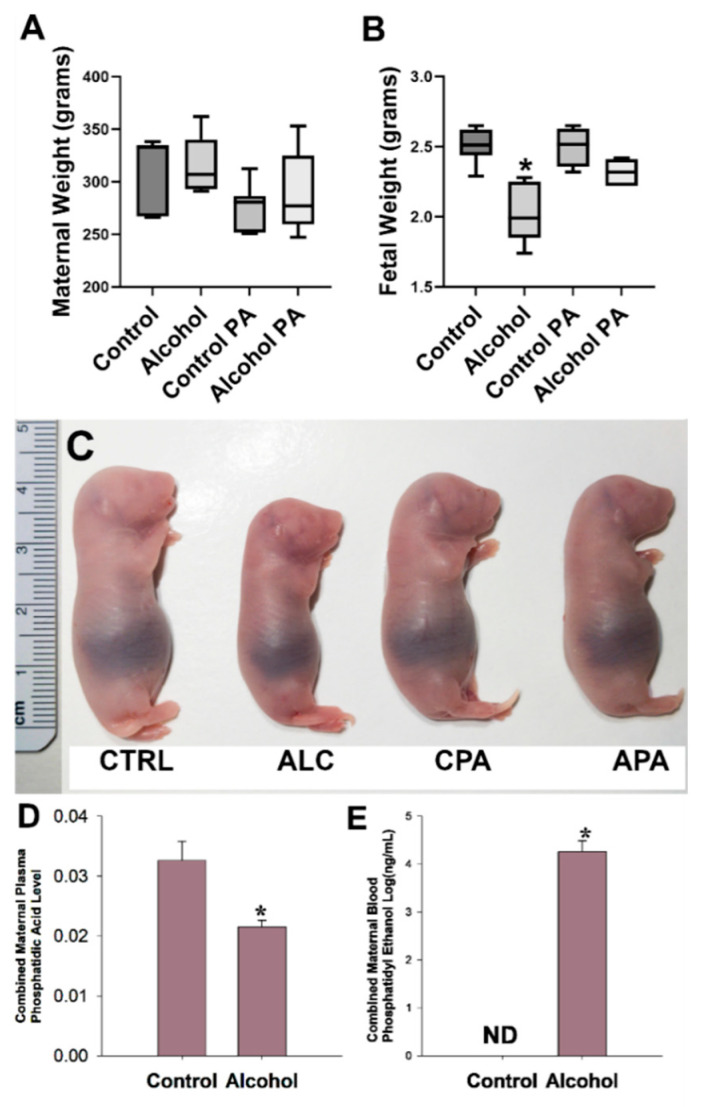
(**A**–**C**) Interaction of prenatal alcohol and in vivo phosphatidic acid (PA) supplementation on fetal growth parameters. (**D**,**E**). Effect of alcohol on total maternal plasma PA and maternal blood phosphatidic ethanol (PEth) levels. Values are mean ± SEM, * indicates statistical significance, *p* < 0.05.

Maternal body weights did not differ among the treatment groups (Figure 1A). Mean fetal weights were significantly lower in the alcohol group compared with those in the control (*p* < 0.0001), however, the fetal weight difference between the alcohol and the pair-fed control group was completely abolished by concomitant in vivo PA administration with alcohol, indicating reversal of classic FASD growth restriction phenotype (Figure 1B). The control PA group was not different to the alcohol PA group. Representative fetuses that are in the median weight range in each of the four treatment groups are depicted in Figure 1C. Litter size among all groups in the cohort were not significantly different (average litter size, control, 10.83 ± 2.56; alcohol, 10.83 ± 1.33; control PA, 10.00 ± 1.54; alcohol PA, 10.00 ± 1.09).

### 3.2. Phosphatidic Acid and Phosphatidyl Ethanol Assessments

Following mass spectrometry, we previously identified subspecies of maternal plasma PA and maternal blood PEth in our model [37,38]. We evaluated the combined abundance of PA (Figure 1D) as well as PEth (Figure 1E) since the combined PEth is a powerful biomarker for alcohol consumption in humans. The total PA level was significantly lower in the alcohol group compared to the controls (*p* = 0.011) and this was accompanied by concomitant increases in levels of total PEth confirming our hypothesis that PEth was formed at the expense of PA as PEth was not detected as expected in the control dams.

### 3.3. In Vivo PA Supplementation and Reversal of Alcohol-Induced Vascular Dysfunction

**Figure 2:**
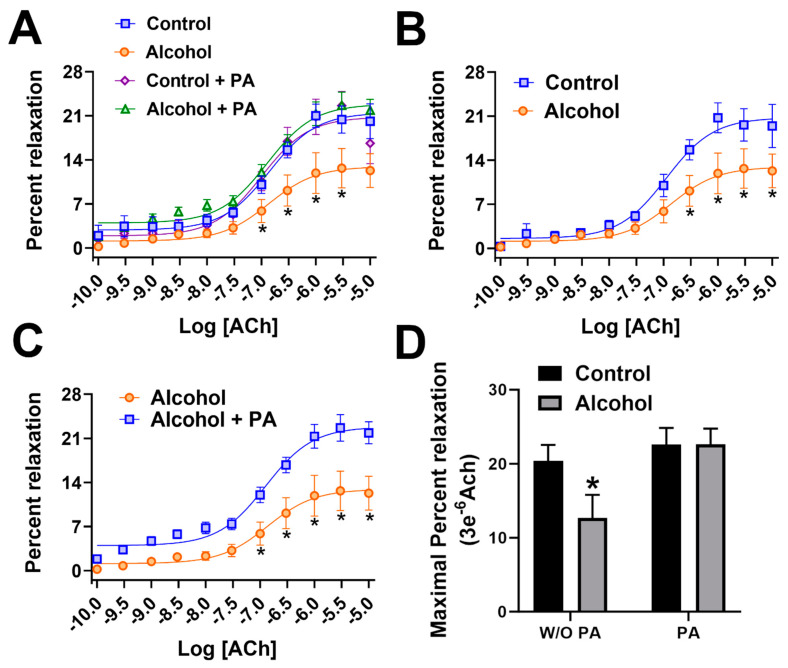
(**A**–**D**) Percent relaxation of rat uterine arteries following acetylcholine. In vivo Phosphatidic acid (PA) reversed alcohol-induced dysfunction of the uterine artery in pregnant rats. Data are expressed as mean ± SEM. Significance (*) was established a priori at *p* < 0.05.

Following in vivo administration of binge alcohol with or without concomitant in vivo PA throughout gestation, vascular function was assessed using pressure arteriography. An interaction of ACh dose X in vivo treatments was noted (Figure 2A). A significant main effect of ACh dose (*p* < 0.0001) and the in vivo treatment (*p* < 0.0001) were also noted. Vasodilation following ACh, an endothelium-dependent agonist, was significantly decreased in uterine arteries of the alcohol group compared with those in the controls (*p* < 0.05, Figure 2B). In vivo supplementation of PA throughout pregnancy abolished alcohol-induced decreases in uterine artery vasodilation (Figure 2C). We administered PA in vivo in the absence of alcohol in the control PA group and we detected no differences in the vasodilation between the control PA and alcohol PA groups (Figure 2A). Maximal ACh-induced vasodilation was significantly different in the alcohol group compared to the control, control PA, and alcohol PA groups (*p* < 0.05; Figure 2D).

### 3.4. Alcohol-Induced Decreases in Stimulatory eNOS Phosphorylation

**Figure 3:**
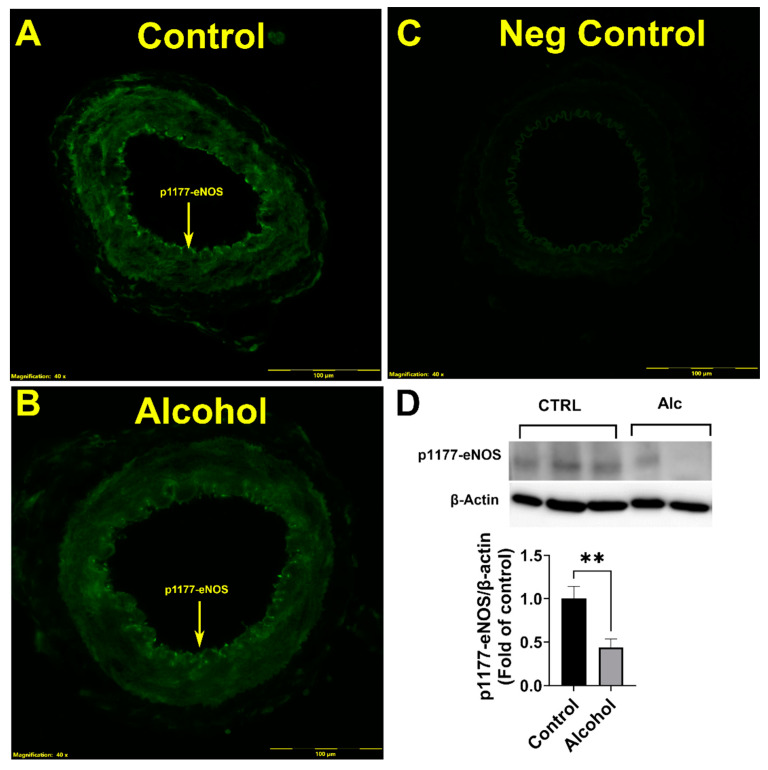
(**A**–**D**) Alcohol-induced decreases in stimulatory p1177-eNOS phosphorylation in the endothelium of the uterine artery. Significance (**) was established a priori at *p* < 0.05.

Immunofluorescence imaging demonstrated that excitatory p1177-eNOS was detected in the endothelium of the uterine artery (Figure 3A–C). The level of florescence showed major decreases in the levels of phosphorylation at the p1177-eNOS, and was validated by immunoblotting analysis. Alcohol significantly reduced (*p* = 0.0362) the proportion of phosphorylated p1177-eNOS in the uterine artery (Figure 3D).

### 3.5. In Vivo Phosphatidic Acid (PA) Reversed Alcohol-Induced Decreases in Stimulatory eNOS Phosphorylation

**Figure 4:**
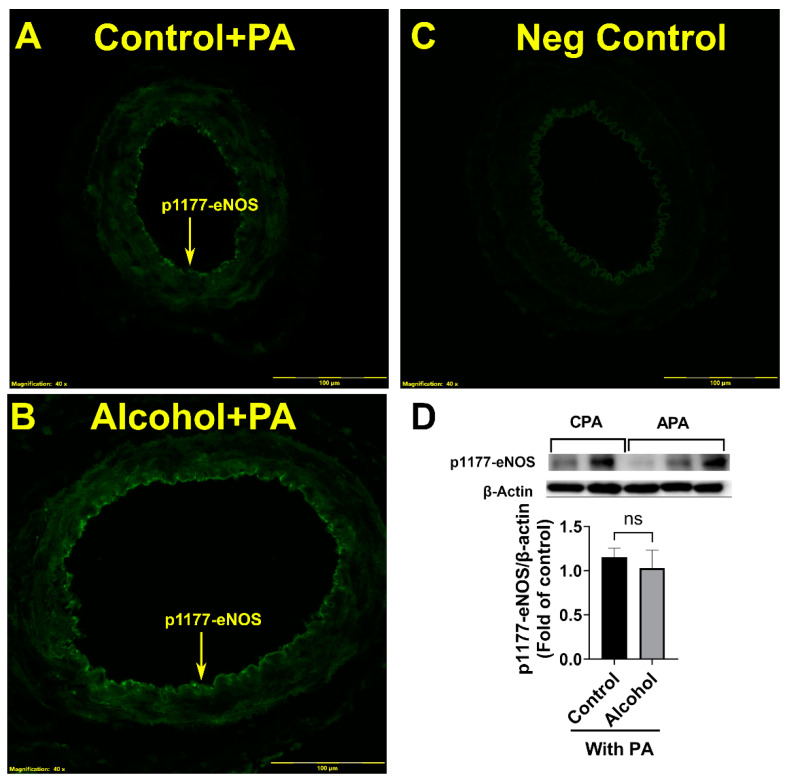
(**A**–**D**) In vivo Phosphatidic acid (PA) reverses alcohol-induced decreases in the uterine artery excitatory p1177-eNOS levels in pregnant rats. Significance as established a priori at *p* < 0.05. ns, not significantly different.

When comparing p1177-eNOS normalized to β actin, the alcohol-induced downregulation of p1177-eNOS was completely reversed following in vivo PA supplementation. p1177-eNOS relative to β actin in the control group was not different between the control PA and the alcohol PA groups (*p* = 0.8122; Figure 4).

**Figure 5:**
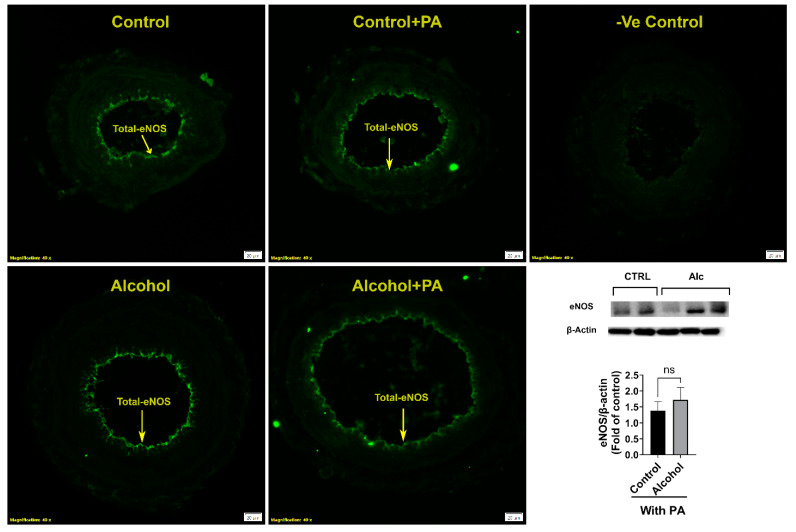
Effect of alcohol and/or in vivo PA on uterine artery total eNOS expression and its localization. Significance was established a priori at *p* < 0.05. ns, not significantly different.

We further confirmed if total eNOS was altered by alcohol (Figure 5). Total eNOS was not different among groups showing that the mechanism of action was due to alcohol actions on p1177-eNOS, a potent excitatory site of the endothelial derived eNOS vasodilatory protein. Neither alcohol nor PA had any effect on total eNOS. Further, immunofluorescence showed that total eNOS was also localized to the uterine artery endothelium.

## 4. Discussion

To date, FASD literature has generally focused on either direct actions or secondary effects of alcohol on the developing brain and the subsequent behavioral adaptations of the offspring. More recently, research has extended to studying the effects of alcohol on miscarriage, stillbirth, preterm birth and sudden infant death syndrome [48]. However, this is not sufficient to explain the growth deficits that stem from prenatal alcohol exposure [49,50]. The current report not only documents alcohol-induced maternal and fetal FASD phenotypes [51] but also identifies etiological factors in FASD. Although the current study identifies a connection between PA and eNOS, and the subsequent rescue of uterine artery dysfunction following gestational alcohol exposure, developing a more comprehensive mechanistic framework that explains the rationale for PA-induced alterations to eNOS system can in the future lead to the creation of effective prevention and/or treatment approaches for FASD.

**Figure 6:**
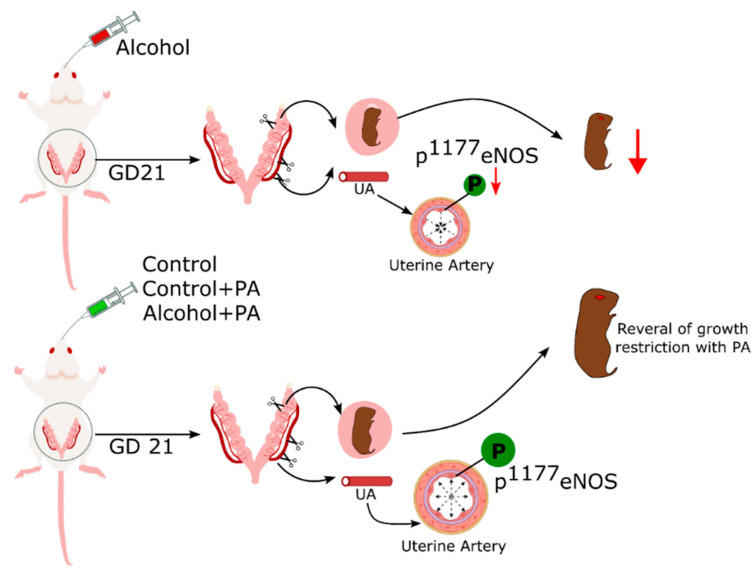
Plausible mechanistic model of interaction between in vivo PA and alcohol in rat uterine artery.

All reported models of intrauterine growth restriction (IUGR) show altered uterine vascular adaptations. IUGR is a cardinal feature of fetal alcohol syndrome. In the current study, we had hypothesized that PA, a molecule directly related to alcohol metabolism, plays a major role in alcohol-induced deficits in agonist-induced vasodilation, eNOS activity and expression, in the maternal uterine artery. The identification of alcohol target molecules is essential for elucidating etiologies of FASD outcomes, which remain poorly understood. The novelty of the reported findings lie in identifying and utilizing a pathway regulating Phosphatidylethanol (PEth; the most promising, 100% specific, and most sensitive biomarker for prenatal alcohol exposure [52]) formation because during alcohol metabolism, phosphatidylcholine undergoes transphosphatidylation to PEth instead of phosphatidic acid (PA, an essential nutrient for growth and development) [53]. Further, we herein report that in vivo PA administration reverses alcohol-induced deficits in uterine artery eNOS activity index; a system reported previously to directly affect alcohol-induced vasodilation in our model and completely rescues IUGR, a major phenotype of FASD (Figure 6).

### 4.1. Development of Lipid-Based Treatment Strategies in Pregnancy

Development and translation of intervention strategies in an obstetric setting has been largely derived from what works in nonpregnant patient populations. A clear mechanistic comprehension of the drug disposition, pharmacologic end effects, and drug targets is required, compounded with an analysis of unwanted end effects from the perspective of the mother as well as the fetus [54]. Interestingly, only nine drugs have undergone the elaborate safety and efficacy testing as required by the Food and Drug administration (FDA) for use in an obstetric setting [55]. These drugs include Methergine, Syntocinon, Pitocin, Yutopar, Cervidil, Magnesium sulfate, Makena, and Diclegis [55]. Reasons for the low number of medications that have been extensively tested could be related to multiple factors, including risk-benefit analysis of the pharmaceutical/nutraceutical drug, a lack of a clear understanding of the great obstetric syndromes, a need for better and early detection of adverse gestational outcomes, and the multi-mechanistic etiologies of gestational conditions [54,55]. The current study takes advantage of an endogenous biomolecule (PA) and strategically manipulates the pathway associated with alcohol metabolism and lipid biosynthesis in pregnancy, effectively preventing at least some of the deleterious effects of prenatal alcohol exposure.

### 4.2. Novelty of Utilizing PA in Discovering Etiology of Alcohol-Related End Organ Effects

PA is a molecule directly affected by alcohol (ethanol) exposure. Alcohol inhibits the formation of PA and is in turn converted to Phosphatidylethanol (PEth) [37,39], which is currently used as a gold standard for alcohol exposure detection in pregnancy [56,57,58]. Although PEth is widely known as a biomarker of alcohol exposure, the mechanistic pathways related to PEth are not well known. Interestingly, we report that combined PEth replaces total PA in the tissues in proportion with the abundance of alcohol in the system [37]. We have reported that several major isoforms of PEth were detected in a number of tissues in our FASD rat model. These tissues include: the uteroplacental compartment, maternal blood, fetal blood, and fetal brain regions (cortex, cerebellum, and the hippocampus) [37]. Concomitant with these increases in PEth levels, the magnitude abundance of the PA isoforms were significantly decreased in our FASD model [38]. The harmful result of a low p1177-eNOS activity index is an impaired ability of a blood vessel to dilate. The current manuscript presents evidence of a link between PA and eNOS, especially in the uterine artery. A possible explanation is that due to its conelike shape, PA can introduce a negative curvature into the membranes on which it has been incorporated, influencing vesicular trafficking as well as altering the topography of the outer lipid membrane, that in turn can alter signaling pathways that work through proteins or lipids residing on the cell membrane such as eNOS [59]. In the case of alcohol exposure, there is a deficit of PA and a concomitant increase in PEth and this dynamic could affect eNOS post-translational modification and subsequent function of the uterine artery. Interestingly, the use of PA as an intervention strategy exploits a mechanistic pathway that is specific to ethanol as the endogenous PA in our tissues reacts with ethanol to form PEth.

### 4.3. Alcohol-Induced Uterine Artery Dysfunction and Its Reversal by PA

Uterine arteries are unique among all blood vessels during pregnancy as their diameter doubles from nonpregnant state to second trimester and triples by third trimester in humans. These adaptations are accompanied by a change in the blood velocity from 8 cm/s in the nonpregnant luteal state to almost 61 cm/s in the third trimester of gestation [60]. Further, the uterine blood flow increases by ~25 fold in the third trimester compared to the nonpregnant luteal phase [14,61]. In summary, gestational uterine vascular adaptations are critical for gas and nutrient delivery and are correlated with fetal growth [11,26,61,62,63,64,65]. Alcohol is shown to have a direct impact on uterine arterial adaptations [26], including endothelium-dependent acetylcholine-mediated uterine artery vasodilation in rats [22,23] and sheep [66], in addition to vessel remodeling at the level of the spiral artery [67]. In vitro alcohol exposure alters the transcriptome [68] and the proteome of the uterine artery [24,69]. In humans, PA supplements of 750 mg per day are consumed for health benefits, though literature in pregnancy is limited [70]. In the current study, we supplemented PA in vivo concomitant with alcohol gavage and demonstrated significant rescue of vasodilatory deficits resulting from alcohol exposure. However, the dams that were not fed alcohol did not exhibit any additional benefit in arterial function following PA supplementation; this is not unexpected, as the anticipated concentration-dependent vessel diameter was already achieved in the control dams. To establish specificity, we previously supplemented ex vivo PA by directly adding the molecule onto the excised uterine artery tissue after isolation at the end of the alcohol treatment paradigm and reported that direct PA addition significantly reversed uterine artery dysfunction [39]. The current study is a significant step to advance FASD research, as the data clearly demonstrates that in vivo PA supplementation along with alcohol would effectively prevent alcohol-induced uterine artery dysfunction. In support of our findings, outside the FASD field, PA has been reported to play a role in arterial function [71,72,73].

### 4.4. PA Reversed Alcohol-Induced Post-Translational Modification of eNOS

We and others have documented alcohol exposure results in impairment of endothelial nitric oxide-mediated vascular function of the maternal uterine artery [17,20,43], including significant decreases in excitatory p1177-eNOS levels. We previously reported that in vitro PA addition directly to the uterine artery reverses alcohol-induced decreases in excitatory p1177-eNOS levels [39]. The current study establishes that in vivo PA administration has a robust preventive effect when it comes to countering alcohol’s negative impacts on uterine artery vasodilation and the vasodilatory eNOS system. It is reported that endothelial nitric oxide agonists have a requirement for optimal levels of PA for nitric oxide production [74]. However, this is the first study showing the direct effect of in vivo PA supplementation on eNOS phosphorylation in uterine arteries of pregnant rats and demonstrates a complete reversal of alcohol’s effects on the excitatory eNOS post-translational modification in the uterine artery.

### 4.5. The Beneficial Effects of PA on FASD Growth Phenotypes

A cardinal feature of FASD is growth restriction. Growth deficits associated with prenatal alcohol exposure are observed among children from birth until at least 14 years of age [49,50]. FASD data spanning from the 1970s to present times from children and from animal studies demonstrate reduced weight, height, and birth head circumference [50,75,76,77,78,79]. Pivotal investigations in the area of the developmental origins of health and disease (DoHAD) illustrate direct relationships between low birth weight and risks for adult-onset altered-physiologic functions including cardiovascular function and its regulation, insulin resistance, and nutrient metabolism [80,81,82]. Thus, alcohol-induced deficits to fetal growth can disturb these body functions leading to several adult-onset diseases. The current study focusing on alcohol’s direct actions on the maternal uterine artery provides a logical and direct explanation for understanding the etiology of FASD growth deficits. Fetal growth, neonatal birth weight, and survival are all directly related to major uterine circulatory adaptations in normal pregnancies [11,12]. Based on alcohol metabolism pathways, we have identified a specific alcohol target (PEth and PA) in the uterine artery of the mother, which principally delivers oxygen and nutrients to the fetoplacental compartment, and we present an innovative pharmacologic targeting approach to prevent FASD growth restriction. While it is hard to prove that alcohol-induced reversal of uterine artery dysfunction directly resulted in the rescue of FASD growth deficit, these are welcome findings to a field where there are no approved treatment strategies.

### 4.6. Perspectives

Since the prevalence of FASD has not declined in the U.S. for decades [83,84,85,86,87,88,89], currently utilized prevention approaches must be supplemented by identifying beneficial FASD therapeutic strategies. At this time, there are no FDA-approved drugs for the direct treatment of FASD [90]. A major reason for this is likely the lack of understanding of the multi-mechanistic actions of alcohol during pregnancy which produce heterogeneous outcomes [51,91]. The novel data from the current study on gestational alcohol consumption elucidates a key role for PA (a molecule directly connected to alcohol metabolism) in maternal vascular adaptations that are necessary for optimal fetal growth and development, and such strategic approaches may provide important insights for the future development of translatable interventions.

## Data Availability

All the processed data is included in the manuscript. Further inquiries can be directed to the corresponding author (J.R.).
